# LiDAR-Based Maintenance of a Safe Distance between a Human and a Robot Arm

**DOI:** 10.3390/s23094305

**Published:** 2023-04-26

**Authors:** David Podgorelec, Suzana Uran, Andrej Nerat, Božidar Bratina, Sašo Pečnik, Marjan Dimec, Franc Žaberl, Borut Žalik, Riko Šafarič

**Affiliations:** 1 Faculty of Electrical Engineering and Computer Science, University of Maribor, Koroška cesta 46, SI-2000 Maribor, Slovenia; suzana.uran@um.si (S.U.); andrej.nerat@um.si (A.N.); bozidar.bratina@um.si (B.B.); saso.pecnik@um.si (S.P.); borut.zalik@um.si (B.Ž.); riko.safaric@um.si (R.Š.); 2 FOKUS TECH d.o.o., Ulica Zofke Kvedrove 9, SI-3000 Celje, Slovenia; marjan.dimec@fokus.si; 3 FANUC ADRIA d.o.o., Ipavčeva ulica 21, SI-3000 Celje, Slovenia; franc.zaberl@fanuc.eu

**Keywords:** LiDAR, robot, human–robot collaboration, speed and separation monitoring, intelligent control system, geometric data registration, motion prediction

## Abstract

This paper demonstrates the capabilities of three-dimensional (3D) LiDAR scanners in supporting a safe distance maintenance functionality in human–robot collaborative applications. The use of such sensors is severely under-utilised in collaborative work with heavy-duty robots. However, even with a relatively modest proprietary 3D sensor prototype, a respectable level of safety has been achieved, which should encourage the development of such applications in the future. Its associated intelligent control system (ICS) is presented, as well as the sensor’s technical characteristics. It acquires the positions of the robot and the human periodically, predicts their positions in the near future optionally, and adjusts the robot’s speed to keep its distance from the human above the protective separation distance. The main novelty is the possibility to load an instance of the robot programme into the ICS, which then precomputes the future position and pose of the robot. Higher accuracy and safety are provided, in comparison to traditional predictions from known real-time and near-past positions and poses. The use of a 3D LiDAR scanner in a speed and separation monitoring application and, particularly, its specific placing, are also innovative and advantageous. The system was validated by analysing videos taken by the reference validation camera visually, which confirmed its safe operation in reasonably limited ranges of robot and human speeds.

## 1. Introduction

Human–robot collaboration (HRC) has become important in industry in recent years, due to the demand for increased flexibility in production environments. In small, and medium-sized, enterprises, particularly, the use of robotic arms has often proved to be uneconomical due to the typically small batch sizes.

The technical specification ISO/TS 15066:2016 [1] provides principles and requirements for the design of HRC applications. One of the four HRC scenarios described therein is called speed and separation monitoring (SSM). The short definition of SSM from this technical specification is also used in this paper: “The robot arm maintains a minimum distance towards the human during the execution of its task to avoid physical contact with the human”. This minimum distance is called the protective separation distance (PSD). It is, by definition, the minimum distance which assures that the robot system has the necessary deceleration capability to stop before colliding with a human in the HRC workspace [2]. The PSD is updated in real time after each robot or human movement. It is important to ensure the PSD is as short as possible to avoid unnecessary deceleration and increase HRC efficiency. The introduction of Industry 4.0 concepts brings newer regulatory safety challenges within industrial intelligent human–robot collaboration, as proposed in [3]. Our paper demonstrates the SSM functionality in an HRC application, realised by augmenting a standard industrial robot arm’s controller with a prototype intelligent LiDAR sensor system.

The PSD calculation and SSM itself can be controlled by the sensory data, mathematical models or, as is most frequently the case, both [4,5,6].

A wide variety of sensors can be encountered in the HRC-related literature, which can be positioned on a robot, on locations with a good view near the HRC workspace, sometimes even on a human. Commonly used sensors include the following: a single camera [7] or several cameras [8], stereo cameras [9], RGB-D visual sensors [10], ultrasonic sensors [11], infrared thermal sensors [12], laser-based technologies, including time-of-flight (TOF) sensor arrays, 3D TOF cameras and 2D/3D light detection and ranging (LiDAR) scanners [13,14,15,16], or different combinations, such as 2D laser scanners and a Kinect RGB-D visual sensor [17], or RGB cameras, a depth camera and a thermal imager [18].

Another possibility, often employed, is to represent a robot arm by a mathematical model. When a kinematic model is used, the geometry of the robot arm is established from its state, provided by its controller [19,20,21,22]. On the other hand, more complex dynamic models represent the motion of the robot arm, as well as the forces acting on it and on the human during the collision [23,24]. Humans can also be modelled if they are static, or if their movements can be learned from a huge number of recorded trajectories by employing Artificial Intelligence and Machine Learning techniques [25,26].

Hybrid approaches combine elements of model-based and sensor-based methods, to provide a balance of accuracy, response time and computational efficiency [27,28]. The method proposed in this paper represents the robot arm with its kinematic model, while humans and other dynamic obstacles are extracted from 3D LiDAR data.

Many SSM methods use motion prediction to improve safety and efficiency further. The positions and postures of a human and a robot in the near future are computed from the known real-time and near-past positions and poses, either represented by a mathematical model [29], or acquired from sensory data [30]. PSD is then computed from the predicted positions and postures, and used to control the robot’s speed in the same manner as the real-time PSD. We use the described traditional approach for human motion prediction, while the prediction of the robot model’s state represents the main novelty of our approach. The idea is to load an instance of the robot programme into the ICS, which then precomputes the future position and pose of the robot arm. The robot’s near-future motion is, thus, not predicted, but computed by its original programme, which assures higher accuracy and improved safety in comparison to the traditional approaches.

To our knowledge, utilisation of a 3D LiDAR scanner in an SSM application and, particularly, its placing in an elevated position at the side of the HRC workspace, are also innovative and advantageous, particularly in addressing the so-called grey zones and, at the least, in offering limited possibility in detecting multiple humans. The existing laser-based SSM solutions use mostly 2D scanning in selected horizontal and vertical planes [13,16], or on-robot sensors [15]. Our sensor, in contrast, is located out of the robot’s reach, captures a 3D point cloud, is stationary, and does not include an inertial measurement unit (IMU), a compass, GPS or any other positioning system.

The heterogeneity of the robot kinematic model (either from the robot controller or from the trajectory precomputed by the ICS) and sensor data to be aligned, prevented us from using generic open-source solutions for geometric data registration, such as [31], so we developed our own data registration method. It is still based on well-known rigid transformations, but the choice of the sensor and testbed required some small, partly innovative, adjustments.

The paper consists of four Sections. The testbed, hardware, validation software and equipment and, particularly, the developed intelligent control system are presented in Section 2. The testing scenarios and validation results are provided in Section 3. The work and results are discussed, disadvantages and limitations stressed, and possible improvements listed in the concluding Section 4.

## 2. Materials and Methods

**Figure 1:**
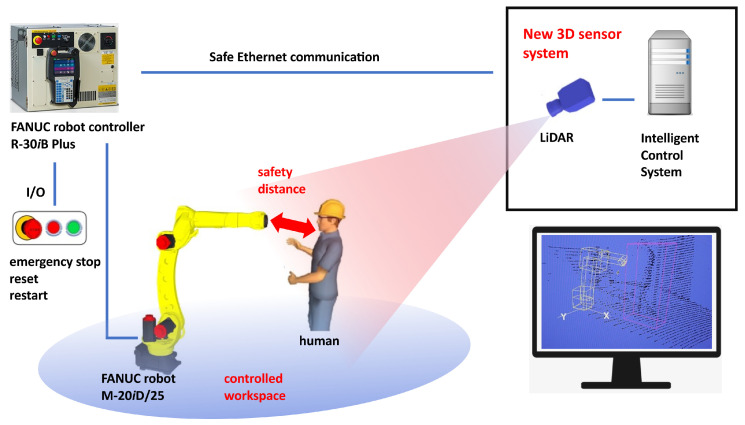
Laboratory setup of a robot application safeguarded with the proposed 3D sensor system.

The presented research aimed at evaluating the performance of a proprietary 3D LiDAR scanner prototype and the associated ICS. The testbed, shown in Figure 1, was set up at the premises of FANUC ADRIA [32]. It includes a FANUC industrial robot arm, the operation of which is controlled in real time by a FANUC robot controller. A human (either an operator or an intruder), an animal, another dynamic obstacle (hereafter, we use the term intruder for all these categories), or a group of them, is also present in the HRC workspace, where he/she/it moves according to pre-prepared relevant scenarios. The workspace is monitored by a 3D LiDAR scanner. The acquired point clouds and the positions of the robot system (the robot arm with a tool and a workpiece), obtained from the robot controller, are processed in real time by the ICS on its associated computer. Although it would be best to place the sensor above the workspace, this is often not possible indoors due to limited height. We placed it out of the robot’s reach, on the side where collision between the robot arm and a human was most likely to occur. We defined the distance so that the sensor could still detect all the reference objects defined in Standard EN IEC 61496-3:2019 [33].

### 2.1. Devices, Materials and Validation Software

**Figure 2:**
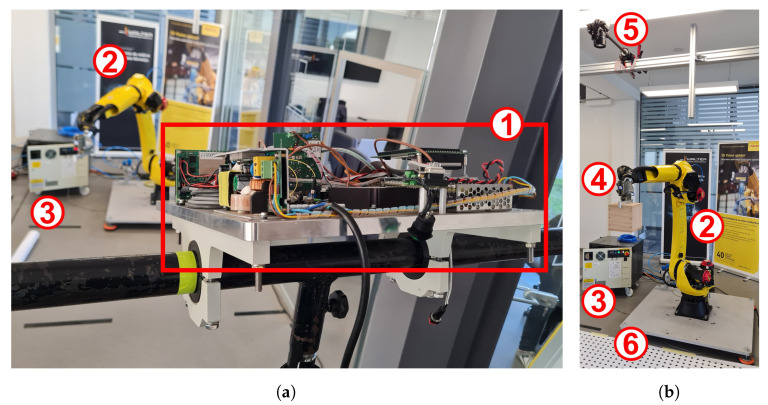
Testbed in the FANUC ADRIA premises, consisting of 1—LiDAR scanner prototype, 2—FANUC M-20iD/25 robot arm, 3—FANUC R-30/B Plus robot controller, 4—metal gripper, carrying a wooden cube, 5—reference validation camera, and 6—polyester canvas with printed grid of dots: (**a**) Test setup (with the permission of FOKUS TECH d.o.o.); (**b**) Validation setup.

A new 3D sensor system represents the core of the proposed SSM application. It consists of a proprietary 3D LiDAR scanner prototype (Figure 2a) developed by FOKUS TECH [34], and the ICS, implemented mostly at the Faculty of Electrical Engineering and Computer Science at the University of Maribor. The employed LiDAR scanner was actually developed for safety and inspection applications on railways [35], and, here, we wanted to validate its usability in some different fields as well. It has an operating range of 10 m, detection (horizontal × vertical) angle $40^{\circ }\times 40^{\circ }$, horizontal resolution of $0.1^{\circ }$ or $0.3^{\circ }$, a vertical resolution of $0.1^{\circ }$ to $0.9^{\circ }$, and an operating temperature range from 5 °C to 40 °C. It operates at 230 V AC with power consumption of 150 W. Table 1 lists the choices of resolutions (transformed into scan lines and samples per line) and scanning speeds in frames per second (fps) available.

The ICS was programmed in C++ and installed on a NanoPi M4V2 single-board computer with a Linux operating system in the presented testbed. The hardware connection between the LiDAR scanner and the ICS computer was by means of an Ethernet cable. The LiDAR scanner pushes the point cloud data in real time as broadcast UDP packets. Every packet contains data for 150 points, along with the timestamp. An EtherNet/IP CIP protocol [36] was utilised for communication with the robot controller. The latter sends the robot joint coordinates and grip status every 8 ms. Commands for robot speed changes are sent in the opposite direction, computed by the SSM or motion prediction ICS modules.

A robot arm, FANUC M-20iD/25 (Figure 2), was used in the described experiments. It was provided by FANUC ADRIA, together with the robot controller, R30iB Plus (Figure 2). It is a 6-joint articulated arm with a slim design (curved J2 arm) to enable greater access, with a 57 mm diameter large hollow wrist, internal cable routing, a reach of 1831 mm, and a load capacity of 25 kg. The FANUC ROBOGUIDE software [37], which was used for verification of the developed kinematic model of the robot arm, was also provided by FANUC ADRIA. The robot arm is equipped with a gripper with metal fingers, which carried a wooden cube with sides of 20 cm and weight 0.5 kg (Figure 2b).

An optical camera and a polyester canvas (3 m × 3 m) with printed dots were also used in the system validation setup (Figure 2b). Due to the relatively low scanning speed of the LiDAR scanner, an ordinary camera was sufficient (we recorded videos with 24 fps and image resolution of 1280 × 720 pixels). In the first experiment, a test dummy was used, while our early experiments with a human intruder included a table as an additional safety barrier between the human and the robot.

### 2.2. Intelligent Control System

**Figure 3:**
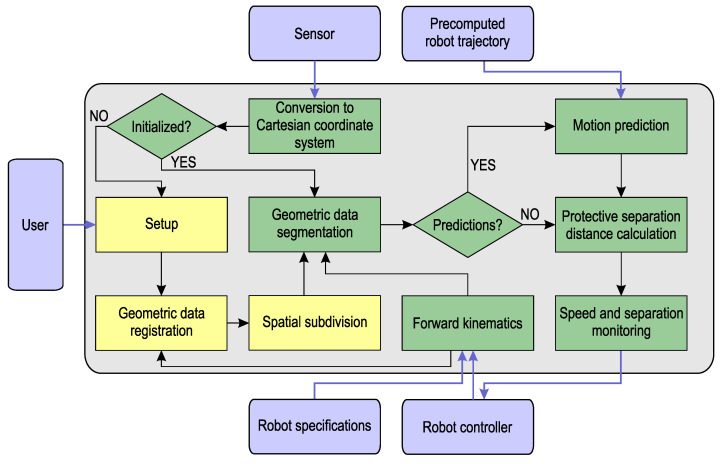
The ICS architecture.

Figure 3 shows the ICS block diagram. The LiDAR scanner acquires a point cloud within a pyramid of vision with viewing angles $40^{\circ }$ horizontally and $40^{\circ }$ vertically. The azimuth and the polar angle of each acquired point are expressed with values in the integer range [0, 4095]. The third point’s coordinate is its distance from the sensor. The optimal location of the sensor is determined according to the size of the HRC workspace and the geometry of the viewing pyramid. It is in the range 3 to 8 m from the workspace. The coordinates of all the acquired points are then converted into the right-handed Cartesian coordinate system of the sensor, such that x∈[MinX,MaxX],y∈[MinY,MaxY] (see Table 1), the *Z*-axis coincides with the central laser beam (x=0,y=0), the horizontal *X*-axis points to the left, and the *Y*-axis is defined by Z×X. The distances are measured in metres. In the initialisation phase, the sensor’s coordinate system and the robot base coordinate system are registered as described in Section 2.2.1. This is followed by uniform spatial subdivision that arranges the LiDAR points into a regular voxel grid [38]. Structured geometric data with simple topology can be processed in a much easier and quicker way than an unstructured point cloud, and, thus, importantly, the subsequent modules are facilitated. Before the registration, the user enters the ICS settings, which include the LiDAR scanning speed, voxel size, the decision as to whether to use a semi-automatic or direct registration method, measurements for the direct registration (if chosen), and the decision as to whether to use predictions (plus the prediction time delay if predictions are used).

The three initialisation blocks are coloured yellow in Figure 3. The other modules (coloured green), which are performed in a loop for each acquired LiDAR frame (the SSM loop in the continuation), are described in separate subsections, except for the conversion to the Cartesian coordinate system, which has already been considered above. The forward kinematics (FK) is described in Section 2.2.2, geometric data segmentation in Section 2.2.3, and motion prediction in Section 2.2.4, while the protective separation distance (PSD) calculation, and speed and separation monitoring are considered in Section 2.2.5.

#### 2.2.1. Geometric Data Registration

The geometric data registration (termed, in short, the registration) is a procedure that determines the transformation that optimally maps two point sets [39]. They are supposed to represent the same scene and must overlap at least partially. In the presented ICS, the registration aims to align the previously time-synchronised LiDAR point cloud and the robot triangulated irregular network (TIN) model [40]. The latter was constructed from the robot arm specifications and put into the appropriate pose by using the robot controller data and the robot’s forward kinematic model (Section 2.2.2). The registration is performed in the application initialisation phase. The results remain valid until the location or orientation of the sensor or robot base changes. The possible slowness of the method is, consequently, not problematic. However, the results are crucial for all further steps of the ICS, and, therefore, a high level of accuracy is required. The registration, thus, uses the highest possible LiDAR resolutions (the first line in Table 1). On the other hand, higher frame rates, and, consequently, lower resolutions are, typically, used in the SSM loop.

The registration task is to find the transformation matrix *M*, which registers points given in the source coordinate system within the target coordinate system. In our case, the former corresponds to the sensor’s local coordinate system $CS_{S}$, while the latter is the robot base coordinate system $CS_{R}$ (referred to as $K_{0}$ in Section 2.2.2). As both $CS_{S}$ and $CS_{R}$ address the same physical space, a reasonable requirement is that they share the same measurement units and the same orientations. Thus, *M* may be considered a rigid transformation, and its content is determined by three translations and three rotations along/around the coordinate axes. The role of registration is to determine the parameters of these six transformations with the best possible accuracy. Several radically different approaches were adapted and tested in the research presented.

Comprehensive reviews of general automatic registration methods can be found, as, for example, in [39,41,42]. Such methods find the transformation by aligning two (or more) geometric data sets, where one is chosen as the target. Two well-rated open-source solutions, PCL [43,44] and TEASER++ [31,45] were tried, but they require strong similarities between the source and target data sets (e.g., a similar number of points, spatial resolution, and points distribution), which our robot TIN model and LiDAR data point cloud could not meet. This task can be facilitated by using some a priori knowledge of the scene, e.g., by first aligning the floor planes (large flat surfaces at the edge of the scene) and then one or more cross-sections parallel to the floor. While this concept is correct, it depends on the input data too much, and is also time inefficient, so we opted for a semi-automatic method rather than automatic ground detection [46,47].

**Figure 4:**
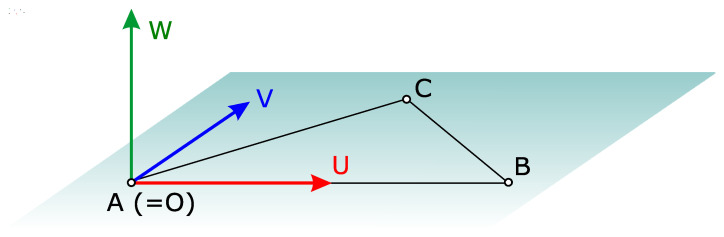
Construction of the intermediate coordinate system from non-collinear points *A*, *B* and *C*.

The semi-automatic method requires the user to select three non-collinear points, *A*, *B* and *C*, interactively in both $CS_{S}$ and $CS_{R}$. The assumption was made here that these two triplets, clicked in two views of a computer-rendered scene, represented the same triplets in the physical world. Furthermore, we assumed that the triplet from the point cloud was also present in the TIN model, but the opposite was not guaranteed. It therefore made sense to select the points in $CS_{S}$ first. This triplet was then used in Equation (1) to establish an intermediate coordinate system $CS_{I}$ with the origin *O* and orthogonal unit coordinate vectors *U*, *V* and *W*, as shown in Figure 4. Then, *M* was computed as a composition of two transformations—$M_{S2I}$ from $CS_{S}$ to $CS_{I}$, and $M_{I2R}$ from the latter to $CS_{R}$. Note that the system of Formula (1), and the interpretation from Figure 4, must be employed separately for $\left\{A_{S},B_{S},C_{S},O_{S},U_{S},V_{S},W_{S}\right\}$ expressed in $CS_{S}$, and $\left\{A_{R},B_{R},C_{R},O_{R},U_{R},V_{R},W_{R}\right\}$ expressed in $CS_{R}$.

(1) $$\begin{array}{c} O=A,\\ U=\frac{B-O}{\left|B-O\right|},\\ W=\frac{U\times (C-O)}{\left|U\times (C-O)\right|},\\ V=W\times U. \end{array}$$

The transformation $M_{I2R}$ from $CS_{I}$ to $CS_{R}$ is given in homogeneous coordinates as the composition of a 3D rotation $Rot_{R}$ and translation $Tran_{R}\left(O_{R}\right)$, as shown in Equation (2):

(2) $$M_{I2R}=Tran_{R}\left(O_{R}\right)\cdot Rot_{R}=\left[\begin{array}{cccc} U_{R}.x & V_{R}.x & W_{R}.x & O_{R}.x\\ U_{R}.y & V_{R}.y & W_{R}.y & O_{R}.y\\ U_{R}.z & V_{R}.z & W_{R}.z & O_{R}.z\\ 0 & 0 & 0 & 1 \end{array}\right].$$

The matrix $M_{I2S}$ from $CS_{I}$ to $CS_{S}$ can be generated in the same manner, but the inverse $M_{S2I}$, as shown in Equation (3), is actually needed:

(3) $$\begin{array}{c} M_{S2I}=M_{I2S}^{-1}=Rot_{S}^{-1}\cdot Tran_{S}^{-1}\left(O_{S}\right)=Rot_{S}^{T}\cdot Tran_{S}\left(-O_{S}\right)\\ =\left[\begin{array}{cccc} U_{S}.x & U_{S}.y & U_{S}.z & 0\\ V_{S}.x & V_{S}.y & V_{S}.z & 0\\ W_{S}.x & W_{S}.y & W_{S}.z & 0\\ 0 & 0 & 0 & 1 \end{array}\right]\cdot \left[\begin{array}{cccc} 1 & 0 & 0 & -O_{S}.x\\ 0 & 1 & 0 & -O_{S}.y\\ 0 & 0 & 1 & -O_{S}.z\\ 0 & 0 & 0 & 1 \end{array}\right]. \end{array}$$

*M* is then obtained by Equation (4) as the composition of $M_{S2I}$ and $M_{I2R}$:

(4) $$M=\left[\begin{array}{cccc} U_{R}.x & V_{R}.x & W_{R}.x & O_{R}.x\\ U_{R}.y & V_{R}.y & W_{R}.y & O_{R}.y\\ U_{R}.z & V_{R}.z & W_{R}.z & O_{R}.z\\ 0 & 0 & 0 & 1 \end{array}\right]\cdot \left[\begin{array}{cccc} U_{S}.x & U_{S}.y & U_{S}.z & 0\\ V_{S}.x & V_{S}.y & V_{S}.z & 0\\ W_{S}.x & W_{S}.y & W_{S}.z & 0\\ 0 & 0 & 0 & 1 \end{array}\right]\cdot \left[\begin{array}{cccc} 1 & 0 & 0 & -O_{S}.x\\ 0 & 1 & 0 & -O_{S}.y\\ 0 & 0 & 1 & -O_{S}.z\\ 0 & 0 & 0 & 1 \end{array}\right].$$

In practice, the user is not able to click two triplets of points in a manner that would ensure they coincided perfectly in the physical world. Consequently, there are two intermediate coordinate systems: $CS_{S2I}$ obtained from $CS_{S}$ through the transformation $M_{S2I}$ from Equation (3), and $CS_{R2I}$ computed from $CS_{R}$ by the inverse of $M_{I2R}$ from Equation (2). The complete transformation is shown in Equation (5):

(5) $$M=M_{I2R}\cdot M_{IS2IR}\cdot M_{S2I}.$$

$M_{IS2IR}$ is generally unknown rigid transformation from $CS_{S2I}$ to $CS_{R2I}$. In Equation (4), the identity matrix is used instead, introducing an error which depends strongly on the user’s skill and precision. A slight improvement can be achieved by assuring congruency of the two triangles formed by the triplets. The $M_{IS2IR}$ remains unknown, but the error is usually reduced in this way.

**Figure 5:**
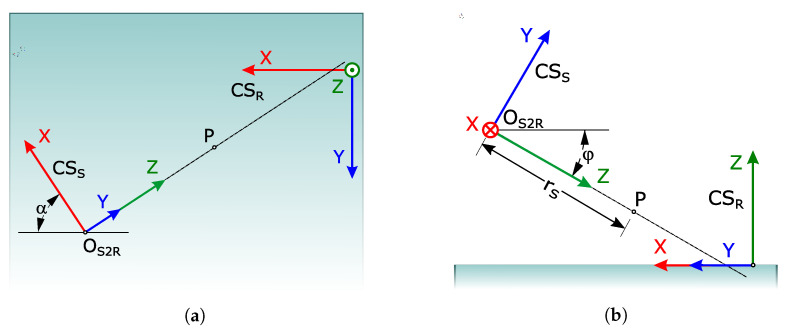
Parameters of the transformation matrix *M* in the direct registration method: (**a**) Top view of the test setup; (**b**) Side view.

Alternatively, the direct registration method can be employed, which determines *M* by measuring the transformation parameters in the physical world. It usually achieves more accurate results than the semi-automatic method, but it requires more operator time and effort. In the considered setup, the *x*-axes of $CS_{S}$ and $CS_{R}$ were both horizontal, which meant that only two rotations were needed. Thus, *M* was determined with 5 parameters: α is the difference between the sensor’s and the robot’s azimuth, φ is the sensor’s inclination, and $O_{S2R}$ is the origin of $CS_{S}$, expressed in $CS_{R}$. However, the sensor is out of reach of the robot arm, and the origin of $CS_{R}$ is hidden inside the robot, disabling direct physical measuring of the coordinate differences between both origins. The problem was solved by using an auxiliary point *P*, which can be determined as $P_{S}$ in $CS_{S}$, and as $P_{R}$ in $CS_{R}$. In the experiment, the sensor’s mirrors were blocked and $P_{S}=\left(0,0,r_{S}\right)$ was, thus, acquired with the central laser beam. A green visible laser was used to enable the robot arm to touch precisely the same point, this time acquired as $P_{R}=\left(P_{R}.x,P_{R}.y,P_{R}.z\right)$. The same central beam could be utilised to determine α, while φ must be measured manually. The entire setup is shown in Figure 5. The transformation matrix *M* is given by Equation (6):

(6) $$M=\left[\begin{array}{cccc} \sin\alpha & -\cos\alpha \sin\varphi & -\cos\alpha \cos\varphi & O_{S2R}.x\\ -\cos\alpha & -\sin\alpha \sin\varphi & -\sin\alpha \cos\varphi & O_{S2R}.y\\ 0 & \cos\varphi & -\sin\varphi & O_{S2R}.z\\ 0 & 0 & 0 & 1 \end{array}\right].$$

The unknown coordinates of $O_{S2R}$ in the last column can be determined from the data acquired by the described measurements using Equation (7):

(7) $$O_{S2R}=\left[\begin{array}{c} O_{S2R}.x\\ O_{S2R}.y\\ O_{S2R}.z\\ 1 \end{array}\right]=\left[\begin{array}{c} P_{R}.x+r_{S}\cos\varphi \sin\alpha \\ P_{R}.y+r_{S}\cos\varphi \cos\alpha \\ P_{R}.z+r_{S}\sin\varphi \\ 1 \end{array}\right].$$

#### 2.2.2. Robot Arm Forward Kinematics

The forward kinematics (FK) of the robot arm determine the positions of the robot tip or robot tool centre point (TCP) in the robot base or world coordinate system for given values of the robot arm joint parameters. The forward kinematic (FK) model serves as a basis to determine the motion and locations of all the links of the robot system. These results are crucial for alignment of the time-synchronised robot TIN model with the LiDAR data in the geometric data registration (Section 2.2.1) and point cloud segmentation (Section 2.2.3) steps, and for subsequent tasks of prediction of the robot’s location (Section 2.2.4) and controlling the robot’s speed with respect to PSD (Section 2.2.5).

**Figure 6:**
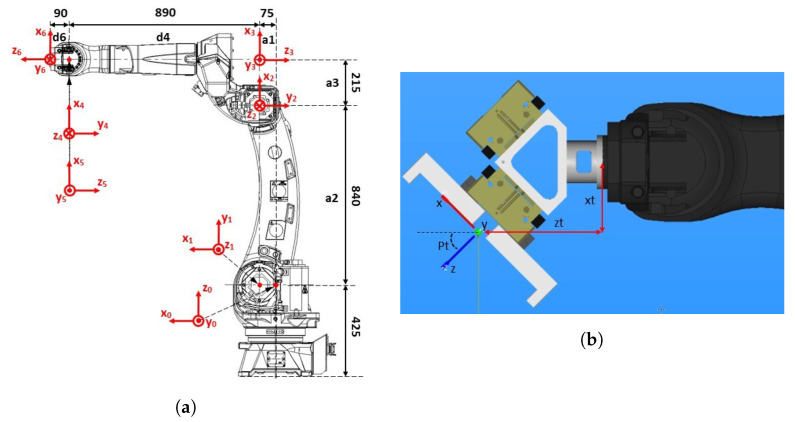
Forward kinematic model of the FANUC M-20iD/25 robot arm: (**a**) Coordinate systems $K_{0}$, $K_{1}$, $K_{2}$, $K_{3}$, $K_{4}$, $K_{5}$, and $K_{6}$ of individual joints (with the permission of FANUC ADRIA d.o.o.); (**b**) The parameters of the transformation $T_{6}^{T}$ between the coordinate systems of the robot tip ($K_{6}$) and the gripper attached to the robot arm.

The FK model for the articulated FANUC M-20iD/25 robot arm was developed using the traditional Denavit–Hartenberg approach [48]. It determines the coordinate systems in a systematic way through a sequence of transformations between them, described by homogeneous 4×4 transformation matrices. The first coordinate system $K_{0}$ is at the robot base and the last one is $K_{6}$ at the robot tip, or $K_{T}$ at the robot TCP. $K_{0}$ to $K_{6}$ are shown in Figure 6a. The intermediate coordinate systems, $K_{1}$ to $K_{5}$, correspond to individual robot joints. The procedure determines $K_{0}$ and the positive directions of the robot joints’ motion, as well as the robot pose and $K_{6}$ (or $K_{T}$) when all robot joints are in a zero position. The transformation matrices $T_{0}^{1}$, $T_{1}^{2}$, $T_{2}^{3}$, $T_{3}^{4}$, $T_{4}^{5}$, and $T_{5}^{6}$ were determined by Equation (8):

(8) $$\begin{array}{c} T_{0}^{1}=Rot\left(z,q_{1}\right)\cdot Tran\left(z,0\right)\cdot Tran\left(x,a_{1}\right)\cdot Rot\left(x,-90^{\circ }\right),\\ T_{1}^{2}=Rot\left(z,q_{2}-90^{\circ }\right)\cdot Tran\left(z,0\right)\cdot Tran\left(x,a_{2}\right)\cdot Rot\left(x,180^{\circ }\right),\\ T_{2}^{3}=Rot\left(z,q_{3}\right)\cdot Tran\left(z,0\right)\cdot Tran\left(x,a_{3}\right)\cdot Rot\left(x,-90^{\circ }\right),\\ T_{3}^{4}=Rot\left(z,q_{4}\right)\cdot Tran\left(z,-d_{4}\right)\cdot Tran\left(x,0\right)\cdot Rot\left(x,-90^{\circ }\right),\\ T_{4}^{5}=Rot\left(z,q_{5}\right)\cdot Tran\left(z,0\right)\cdot Tran\left(x,0\right)\cdot Rot\left(x,-90^{\circ }\right),\\ T_{5}^{6}=Rot\left(z,q_{6}\right)\cdot Tran\left(z,-d_{6}\right)\cdot Tran\left(x,0\right)\cdot Rot\left(x,-90^{\circ }\right), \end{array}$$

where the following values of the Denavit–Hartenberg parameters were used: $a_{1}$ = 75 mm, $a_{2}$ = 840 mm, $a_{3}$ = 215 mm, $d_{4}$ = 890 mm, and $d_{6}$ = 90 mm (see Figure 6a).

Equation (9) gives the FK model of the robot arm without a tool:

(9) $$T_{0}^{6}=T_{0}^{1}\cdot T_{1}^{2}\cdot T_{2}^{3}\cdot T_{3}^{4}\cdot T_{4}^{5}\cdot T_{5}^{6}.$$

This model must be equivalent to the FK model implemented within the FANUC robot controller. This was verified by the FANUC robot simulation software ROBOGUIDE [37]. Numerous repertoires of joint position values $q_{1}$ to $q_{6}$ from Equation (8) were used. A perfect match was confirmed between $K_{6}$, determined by ROBOGUIDE, and $K_{6}$, calculated with our FK model.

In our test setup, the robot arm manipulated a load with the shape of a cube, so it was equipped with an appropriate gripper. Therefore, the robot tool coordinate system $K_{T}$ was added to the FK model, as shown in Equation (10):

(10) $$\begin{array}{c} T_{0}^{T}=T_{0}^{6}\cdot T_{6}^{T},\\ T_{6}^{T}=Tran\left(x,xt\right)\cdot Tran\left(y,yt\right)\cdot Tran\left(z,zt\right)\cdot Rot\left(z,Rt\right)\cdot Rot\left(y,Pt\right)\cdot Rot\left(z,Wt\right). \end{array}$$

Here, xt=−92.998 mm, yt=0 mm, zt=165.123 mm, $Rt=0^{\circ }$, $Pt=-45.0^{\circ }$, and $Wt=0^{\circ }$ are coordinates and the Roll, Pitch, Yaw angles of the robot tool with respect to the coordinate system $K_{6}$. Their meaning is explained in Figure 6b.

#### 2.2.3. Geometric Data Segmentation

Before the ICS calculates distances between the robot and any intruders, these latter must be found in the LiDAR point cloud. This is the role of geometric data segmentation, which is performed after the LiDAR coordinate system $CS_{S}$ has been aligned with the robot’s $K_{0}$ and the controlled HRC workspace voxelised. The segmentation operates in two phases.

**Figure 7:**
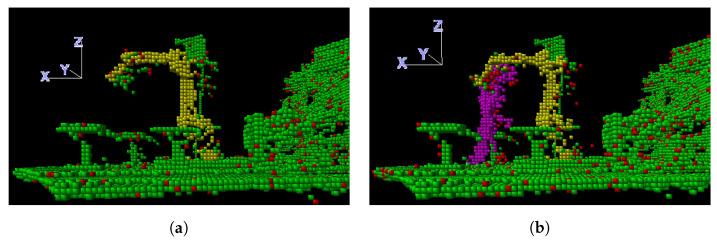
Data segmentation with classification of material voxels representing the robot (yellow), static obstacles (green), intruders (magenta), and noise (red): (**a**) Initial scene without intruders; (**b**) An intruder enters the initial scene.

The robot is first extracted from the initial scene, which is expected to be free of intruders. The voxels within the bounding boxes of the robot’s parts represent the robot, while the remaining material voxels represent either static obstacles or noise. Here, a voxel is considered to be a material voxel if it contains at least one LiDAR point, otherwise it is an empty (air) voxel. This initial segmentation, free of intruders, may also be repeated for different FK-controlled robot poses, to detect eventual material voxels obscured by the robot in previous positions. The static obstacles do not change till the end of the robot programme. Figure 7a shows the material voxels classified as the robot (yellow), static obstacles (green), and noise (red). The latter includes connected areas consisting of non-robot material voxels, the number of which is below a selected threshold (e.g., 5). Note that green voxels, and, thus, static obstacles near the robot tip belong to the gripper and load, which were not included in the kinematic model when these images were captured.

In subsequent frames, the robot is identified in the same way, although its pose might be changed in accordance with FK. Furthermore, the static obstacles remain the same as in the initial frame and can simply be neglected, as the detection of distances between the robot and the static environment has to be provided by the robot’s software and not by the ICS. What is left is noise and eventual intruders. The smaller connected segments (below the threshold) correspond to the former, and the bigger ones represent the intruders. The latter are coloured magenta in Figure 7b.

#### 2.2.4. Motion Prediction

In the presented research motion prediction aimed to assess locations of observed objects in the near future (after $t_{sensor}$, which is the time interval between two consecutive sensor frames) by using the real-time and near-past locations of the observed objects, and to utilise the obtained results to improve the PSD computation (Section 2.2.5). Furthermore, the times of predictions were synchronised with the times of completing the acquisitions of individual sensor frames, which meant that the next frame was actually predicted exactly at the moment when the acquisition of the current one was completed. The use of the motion prediction is optional, as seen from the ICS concept in Figure 3. We further required that the motion prediction method was fast enough (and, consequently, relatively simple, as it was executed periodically at the sensor frame rate) to keep the whole ICS running in real time. There were two types of moving objects that needed to be considered in our ICS: robot links and intruders. A single intruder was predicted in the current solution.

Prediction of the intruder’s location considers the following situations:

1. The intruder has just been detected, and is thus present in a single frame only. The prediction is that he or she is moving directly towards the robot with a standard fast walking speed of 1.6 m/s [49];
2. There are already two consecutive frames containing the intruder. The constant speed of the intruder between the two frames is computed. The prediction is that the intruder continues motion with unchanged speed in an unchanged direction;
3. There are already three or more consecutive frames containing the intruder. The intruder’s locations from the last three slides are used to assess trends in how the speed and motion direction are changing. These trends are then used in predicting the future position. The intruder’s trajectory in this case is a quadratic Bézier curve, i.e., a parabolic arc.

The robot’s positions can be predicted in the same way, but it makes sense to take advantage of the fact that its motion is programmed. Although the robot controller cannot provide the ICS with the robot’s future coordinates in real time, the robot’s programme and, consequently, the trajectories of its links, are, of course, known before the collaborative work starts, and can be provided to the ICS in advance. For this purpose, we used a simple programme written in KAREL (Pascal-based programming language for FANUC robots) [50], which enveloped the actual robot’s programme and recorded all the internal robot parameters with a chosen time step (8 ms, which corresponded to the frequency of sending the robot controller’s data to the ICS) in a single file. This recording was, thus, performed at realistic robot speeds (set in the robot programme) in the initialisation phase, before the ICS started the SSM operation. This meant that the robot links’ locations were actually not predicted, but read, from the list of previously computed locations. The ICS, thus, “predicts” the robot’s joints’ positions in the following manner:

1. The robot controller reports the current robot joint coordinates. ICS uses FK to translate them into the positions of the corresponding links;
2. The ICS must synchronise the real-time trajectory and the stored one. In the described setup, we used a very limited repertoire of the robot’s velocities (0%, 50%, 80% or 100% of the original speed from the robot programme), so ICS was able to determine how many stored positions should be skipped from the current one simply;
3. In the same manner, the recorded positions may be skipped to reach the “predicted” position at a selected future moment.

Note that, at the scanning speed of 4.8 fps, the time interval $t_{sensor}$ corresponded to 26 recorded trajectory positions at full robot speed, 13 positions at 50% speed, and 20.8 positions at 80% speed. Consequently, the latter required interpolation between the 20th and 21st positions.

Motion prediction, realised in this way, is particularly useful if the relative speed of the robot towards the human, or vice versa, is increasing faster than it was before the previous scan frame was processed. This happens when the amplitude or direction of the velocity increases, or the robot and the human simply approach each other in an oblique, rather than a frontal, direction.

#### 2.2.5. Speed and Separation Monitoring

The protective separation distance (PSD) is, by definition, the minimum distance which assures that the robot system has the necessary deceleration capability to stop before colliding with an intruder in the HRC workspace [2]. The PSD depends on the robot’s speed and the intruder’s speed, and their directions at the moment of observation. Consequently, it changes all the time. Generally, it is computed separately for all pairs of movable parts (robot link/tool/load, intruder’s part), but we considered the intruder as a single rigid body. The PSD is computed by Equation (11), described in [1]:

(11) $$PSD=S_{H}+S_{R}+S_{S}+C+Z_{R}+Z_{D},$$

where $S_{H}$, $S_{R}$, and $S_{S}$ represent the intruder’s change in location, the robot system’s reaction time, and the robot system’s stopping distance, respectively. *C* is the intrusion distance safety margin based on the expected human reach. $Z_{R}$ is the robot position uncertainty, which can be negligible due to its small amount (typically 0.1 mm), and $Z_{D}$ is the intruder position uncertainty (e.g., due to point cloud registration error, voxelisation, and low scanner resolution). We used a simplified Equation (12), where $S_{H}$, $S_{R}$, and $S_{S}$ are replaced with the robot and intruder speeds:

(12) $$PSD=\left(v_{r}+v_{h}\right)\cdot \left(t_{sensor}+t_{ICS}\right)+\left(\frac{v_{r}}{2}+v_{h}\right)\cdot t_{stop}+C+Z_{R}+Z_{D},$$

where $v_{r}$ is the robot system’s speed towards the intruder, $v_{h}$ is the intruder’s speed towards the robot system (maximal value 1.6 m/s), $t_{sensor}$ is the time between two sensor scans, $t_{ICS}$ is the delay caused by ICS data processing, and $t_{stop}$ is the robot’s smooth stopping time after the stop command is received. We used $t_{stop}=0.512$ s, which is the smooth stop time for the FANUC M-20iD/25 robot at the highest robot arm tip speed 2 m/s and the maximal load 25 kg. However, the actual stopping time in our tests with fifty times lighter load and lower robot tip speed was significantly shorter (which increased the chance for false positives, but did not affect the false negatives).

Each robot link is represented by the corresponding object-aligned bounding box (aligned with the axes of the local coordinate system of the considered robot joint), while a vertical cylinder, called a safety buffer, is used for the intruder. It has the height of the intruder, extended at the top by half of a voxel’s side *d*. Its central axis CA goes through the centre of gravity computed for the set IV of the intruder’s voxels, and its radius *r* is the dynamically-updated distance from CA to the most distinct voxel *v* from IV, extended by half of a voxel’s side diagonal, as shown in Equation (13):

(13) $$r=\max_{v\in IV}\left(distance\left(v,CA\right)\right)+d\cdot \frac{\sqrt{2}}{2}.$$

The concept of PSD is closely related to the SSM principle. SSM prescribes that the speed of the robot system must be related to PSD, so that, at any time, the robot has the necessary deceleration capability to achieve a complete stop before coming into contact with an intruder, despite the fact that the intruder is moving towards the robot arm [2]. Due to the limited repertoire of robot speeds used, the optimal speed can be selected easily by checking all the choices and selecting the optimal one. The SSM algorithm is as follows:

1. Determine PSD for the current situation for all four robot speeds.
2. Set D= distance between the robot and the intruder.
3. Choose the maximum PSD below *D* and set the $v_{r}=$ robot speed related to that PSD.
4. If (Predictions are used) then
  ▹ Predict the positions after time Δt by using the robot speed $v_{r}$.
  ▹ Determine PSDs for the predicted situation for robot speeds not exceeding $v_{r}$.
  ▹ Set D= distance between the predicted positions of the robot and intruder.
  ▹ Choose the maximum PSD below *D* and update $v_{r}$ accordingly.
5. Send $v_{r}$ to the robot controller.

### 2.3. Validation of the Protective Separation Distance Calculation

**Figure 8:**
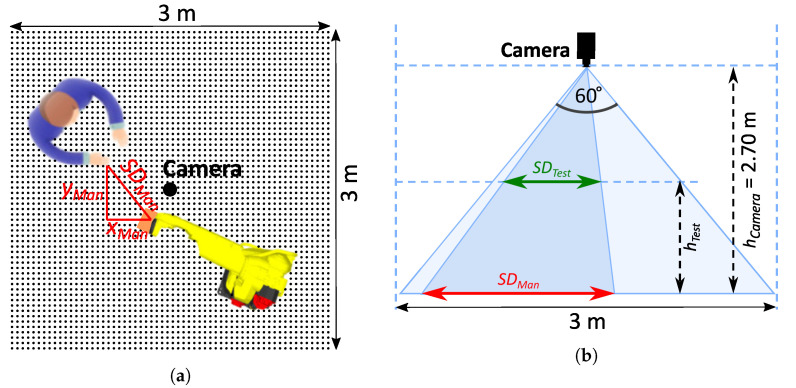
Validation camera setup: (**a**) Top view; (**b**) Side view.

We tested the SSM functionality of our ICS in several test scenarios (Section 3.1) with different parameter values. However, self-validation cannot ultimately confirm the performance and safety of the system. An additional validation was needed using a reference measuring device. We decided to carry out tests using the COVR ROB-MSD-3 safety protocol [51], which we were involved in developing in the past. An optical camera was mounted at a height of $h_{Camera}$ m above the part of the workspace where the most intensive HRC activity was expected. It recorded videos with 24 fps and an image resolution of 1280 × 720 pixels. Canvas with a grid of dots was spread on the floor. The dot diameters were 2 cm and the spacing between the centres of the dots was 5 cm. By counting the dots, we determined the distances $x_{Man}$ and $y_{Man}$ between the robot and the intruder in two horizontal coordinate directions, from which we then calculated the Euclidean distance $SD_{Man}$ (Figure 8a). The label Man stands for “measured manually”. However, HRC action does not usually take place on the ground, where $SD_{Man}$ was measured. The latter was, therefore, only a projection of the actual distance $SD_{Test}$ at height $h_{Test}$. This distance was calculated using Equation (14), and is illustrated in Figure 8b.

(14) $$SD_{Test}=\frac{h_{Camera}-h_{Test}}{h_{Camera}}\cdot SD_{Man}.$$

**Figure 9:**
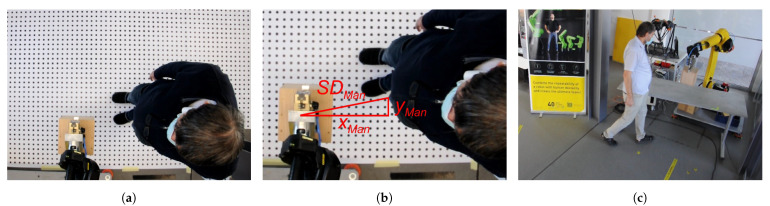
Testing and validation: (**a**) An image acquired from the validation camera video; (**b**) Counting dots in the validation procedure; (**c**) A human intruder approached the robot arm in test scenario 1.

The idea of the validation test was simple. After the robot arm stopped, the intruder also immediately stopped to avoid a collision. It was important that the intruder’s speed remained unchanged till this moment. The recorded video was then processed off-line (Figure 9a,b). $SD_{Test}$ was determined from the first frame after the robot stopped. It was then compared with PSD from Equation (12). In fact, we could use a simplified form, Equation (15), since $v_{r}$ and $v_{h}$ were both 0 after the stop. The test was successfully passed if $SD_{Test}>PSD$, which meant that the robot and the intruder stayed far enough apart that the PSD was not violated.

(15) $$PSD=C+Z_{R}+Z_{D}.$$

Equation (15) suggests that the validation criterion could be satisfied trivially by choosing low values for the user parameters *C*, $Z_{R}$, and $Z_{D}$. However, these values were not only for validation purposes, but primarily to ensure the functionality of the SSM by the ICS. They could only be changed by the user during the ICS initialisation phase, while the ICS had the possibility to affect the PSD calculation during the operation by adjusting $v_{r}$ in Equation (12) only. The intruder’s speed $v_{h}$ could also change dynamically, but the ICS had no impact on it.

Note that the ICS could also be validated additionally, or alternatively, by measuring the time $t_{stop\_Man}$ taken by the robot from the start of braking to a complete stop. For this purpose, the last two video frames should be identified, in which the robot still moves at nominal speed. The validation test was passed when $t_{stop\_Man}<t_{sensor}+t_{ICS}+t_{stop}$.

## 3. Results

### 3.1. Test Scenarios

We identified four scenarios which addressed all possible types of collisions between the robot system and a human intruder in a controlled HRC workspace practically.

- Scenario 1—Slow movement of the intruder towards the robot. The robot arm carried a cube-shaped load with the tip moving at a speed of 0.2 m/s towards the intruder. The latter was moving towards the HRC workspace with a speed of approximately 0.4 m/s. When they became close to each other, the speed of the robot first decreased, and then it stopped completely. The intruder then moved away from the robot, and the latter started to move again (Figure 9c, Supplementary Video S1).
- Scenario 2—Fast movement of the intruder towards the robot. The scenario was similar to the previous one. Here, the speed of the intruder approaching the robot arm was approximately 1.6 m/s (Supplementary Video S2).
- Scenario 3—The intruder was standing in the HRC workspace and the robot arm was moving towards the intruder. This scenario extended the previous two, with a case where the speed of the intruder was zero (Supplementary Video S3).
- Scenario 4—The intruder approached the HRC workspace with his hand only. This scenario demonstrated that the ICS also responded to movements of the intruder’s body parts, not just to his walk. When the intruder’s arm moved away from the robot arm, then the robot programme continued (Supplementary Video S4).

In all four scenarios, the robot stopped safely and avoided a collision with the intruder. However, there were situations where the distance between the stopped robot and the intruder was very close, certainly below the PSD. An analysis is done in Section 3.2.

### 3.2. Validation Results of the Protective Separation Distance Calculation

Table 2 shows the measured distances and validation results for 12 examples. They are documented in the Supplementary Videos S5–S16. In all the examples, the robot’s tip with the gripper and load was moving towards the intruder with $v_{r}=0.2$ m/s. The intruder was moving towards the robot with $v_{h}=0.4$ m/s in examples 1–5 (Scenario 1), standing in front of the moving robot (examples 6–8, following Scenario 3), or approaching the robot with his hands only (examples 9–12, Scenario 4). In case 12, exceptionally, the intruder was holding a cushion. The directions of movement varied from case to case, which is reflected in the rather heterogeneous $x_{Man}$ and $y_{Man}$ values. The test parameters were: $h_{Test}=1.15$ m, $h_{Camera}=2.70$ m, C=0.1 m, $Z_{R}=0.0001$ m, $Z_{D}=0.1$ m, $t_{stop}=0.512$ s, $t_{sensor}=0.2$ s, $t_{ICS}=0.2$ s, and the length of a voxel’s side was d=0.1 m.

Note that the load was not included in the FK model. TCP was, thus, considered in the measurements, as shown in Figure 9b. Furthermore, we ignored the intruder’s feet, which were not relevant for the experiment. Consequently, one or two lower voxel layers were not taken into account in Equation (13), when calculating the intruder’s safety buffer.

In all five considered examples of scenario 1, the test was comfortably passed. However, the results in the column $SD_{Test}$ were scattered between 21.1 and 31.9 cm. This was due, mostly, to the fact that the ICS computed the safety buffer (Equation (13)) in the voxel space, while the measurements were performed in the physical space. The distances between the robot and the intruder at the start of the robot braking could, for example, differ by $d\cdot \sqrt{2}\approx 14.1$ cm.

The lower deviations of the $SD_{Test}$ from PSD in Scenario 3 were due to the fact that, here, the intruder was facing the robot with his shoulder, which was indeed his closest point to the robot. Namely, he usually approached the robot frontally in scenario 1. The test failed in example 7, which was due to the violation of the safety margin (see Section 4).

As expected, we encountered major problems in the validation of Scenario 4. The test failed in cases 10 and 11, where the fingers of the intruder’s left hand were approaching the robot slightly in front of the right hand, making the detection unreliable.

The prediction mode was used in all 12 cases. However, the robot’s speed was actually reduced by the prediction only in cases 8, 9, 11 and 12, where the human and the robot were approaching each other obliquely. In case 8, the prediction actually prevented the PSD violation. In cases 9 and 12, the human had already stopped while the robot was braking, so the prediction was not decisive. In case 10, the human’s hands were not detected at all, so there was a violation of the PSD. In case 11, the hands were detected too late, when even the predictive mode did not help anymore.

Note that the description of the validation protocol [51] also presents a case of Scenario 2 where only 6 of 10 iterations passed the test. Higher speeds would require a higher sensor frame rate to enable the robot to start braking earlier.

### 3.3. Protective Separation Distance Calculation

Of course, we were not able to validate the methods and test setups of other authors with the described protocol. However, we managed to obtain data to compare the PSD, calculated by our method, with those of two reference methods, on a few selected pairs $\left(v_{h},v_{r}\right)$. The results are shown in Table 3. Although the two reference methods involved different robots, safety sensors and test setups, the results were, nevertheless, comparable. To stop safely, our robot can, in most cases, start braking at a very similar distance from the human as the robot in the reference method [12] from 2021. In the latter, *C*, $Z_{R}$ and $Z_{D}$ were completely ignored, otherwise the results would be even closer together. The calculated PSD in the slightly older method [13] from 2012 was mostly longer.

## 4. Discussion

A new hybrid method for speed and separation monitoring in human–robot collaboration applications was introduced in the paper. It aligns, peridocally, the geometric data acquired by a LiDAR scanner and an FK-controlled TIN model constructed from the robot specifications and real-time positions obtained by the robot controller. The aligned datasets then serve for scene segmentation, PSD computation and SSM. The presented system passed tests successfully in four realistic scenarios. Furthermore, it was validated against the COVR ROB-MSD-3 safety protocol [51], demonstrating that the tested LiDAR scanner has satisfactory performance to provide real-time HRC workspace safeguarding in reasonably limited ranges of the robot’s and intruder’s speeds. However, the employed scanner, the ICS, and test setup are still in the prototype phase only. Let us conclude the paper with some further clarifications on the current results, and possible improvements and adjustments to the risk assessments of potential applications. When the validation test reports Failed, the utilised safety protocol [51] prompts the tester to try to identify the reasons for the test failure. These reasons may be the following:

- Grey zones. A grey zone is an area in the HRC workspace which cannot be safeguarded all the time due to obstacles between the sensor and this area.
- Inability to detect narrow objects. A human arm is a reference object, requiring that two scanning rows or columns at the operational distance should not be more than 5–6 cm apart [51].
- Safety margin violation. This critical situation can arise if an intruder suddenly appears from a grey zone, or is already present close to the robot when the ICS activates the SSM.
- Low sensor scanning speed. If $t_{sensor}$ is too long, the speed and direction of movement of the human or robot may change in two consecutive sensor frames in such a way that it is no longer possible to stop the robot in time.
- “Out-of-range” $v_{r}$, $v_{h}$ and/or weight of the load. The validation confirmed the safe operation in reasonably limited ranges of robot and human speeds. The maximum $v_{r}=2$ m/s and load capacity of 25 kg were given in the robot arm FANUC M-20iD/ 25 specifications. However, the maximum $v_{h}$ was not defined strictly, and depended on the physical limitations of the individual. PSD=2.971 m at the standard fast walking speed $v_{h}=1.6$ m/s, $v_{r}=2$ m/s (see Table 3) and load of 25 kg ensured that the robot arm with a reach of 1.831 m would stop at least 32 cm from a human, which is outside the required minimum PSD=20 cm (Table 2). On the other hand, human speeds above 1.6 m/s do not guarantee safe operation, as the calculated PSD is often above the dimensions of the workspace, which usually results in safety margin violation.

In future work, it is also worth addressing the following challenges that were not detected directly by the validation procedure:

- Advances in models and calculations. The distances from each robot’s voxel to the closest intruder’s voxel could be found easily, but this calculation would increase $t_{ICS}$ significantly. Therefore, only the local coordinate systems’ origins of the robot’s joints are considered in the current version of the ICS. The tests in Section 3 were accelerated, additionally, by considering only TCP (see Figure 6b and Figure 9b and Equation (10)), which was indeed the closest to the intruder in most cases. Furthermore, circular moves of the robot links were interpolated linearly. The error compensation was included in the parameter *C*.
- False negatives. Detection of false negatives usually occurs when the calculated PSD is too high, due to the oversizing of individual parameters. Some of these cannot be determined accurately. For example, we used $t_{stop}=0.512$ s, which corresponds to the worst-case value (at the highest $v_{r}$ and heaviest load). It is highly important to estimate such parameters in a manner to increase the PSD and not to decrease it. The efficiency may be sacrificed for the good of safety, while the opposite is not allowed. Note that false negatives can also be met during validation, due to the estimation that the intruder and the robot are operating at approximately the same $h_{Test}$.
- Multiple intruders. Two intruders forming a connected voxel region are identified as one. The number of intruders detected may vary through time as they move closer or further apart. This makes tracking impossible. As a consequence, the prediction mode is only useful in situations with a single intruder. Particularly dangerous are situations where an intruder suddenly appears from a grey zone behind another intruder.
- Motion prediction. The predictions improve safety slightly by forcing the robot to brake earlier and preventing it from accelerating too soon. They can also make the robot’s operation smoother and more efficient. With the current sensor capabilities, a single prediction one frame ahead is acceptable. In general, however, intermediate predictions in the interval between two frames and predictions several frames ahead could also be useful, depending on $t_{sensor}$, $t_{ICS}$, $v_{r}$, and $v_{h}$.

The following modifications need to be considered to address the above problems and challenges in the future:

- An overhead LiDAR scanner and/or multiple scanners represent the only reasonable way to address grey zones. This approach can also significantly improve, or even enable, the detection of multiple intruders when they are not too close to each other. The registration of data from multiple sensors is conducted in the initialisation phase. Therefore, only a slight extension of $t_{ICS}$ is expected, due to the merging of segmented point clouds. Of course, the sensors must be synchronised, as the point clouds to be merged are assumed to be acquired at the same time. In addition, each sensor must be able to distinguish its own reflected laser beam from beams from other sensors. Wearable sensors are a possible alternative, but represent too large a deviation from the presented concept.
- Higher sensor resolution would improve detection of narrow objects.
- Safety margin violation can be addressed partly, together with grey zones. Besides this, the detection of potential intruders is required before the robot is started, which places additional requirements on the synchronisation of the robot and sensors.
- Higher sensor scanning speeds means a simple replacement of the presented prototype LiDAR scanner with some off-the-shelf product. The increased frame rate would make changes between two consecutive frames more predictable. Consequenlty, a higher $v_{h}$ could be allowed, if reasonable in the limited workspace dimensions. Furthermore, a higher scanning speed is also a prerequisite for advanced intermediate and multiple predictions. Finally, the commercial LiDAR scanners typically have an integrated IMU that could, importantly, unprove the accuracy of the proposed direct registration method (Section 2.2.1).
- Improved specifications of the system parameters could reduce the number of false negatives detected.
- Most of these modifications would increase $t_{ICS}$ and, consequently, require a more powerful computer. The latter would also enable the use of advanced models and calculations.

## Figures and Tables

**Table 1:** Resolutions and frame rates of the prototype LiDAR scanner.

Samples per Line	Scan Lines	MinX	MaxX	MinY	MaxY	Frame Rate
293	292	−146	146	−146	145	0.4
142	141	−71	70	−70	70	1.6
142	70	−71	70	−35	34	3.3
142	47	−71	70	−23	23	4.8

**Table 2:** Validation test results for 12 examples following different scenarios.

Example	Scenario	Video	Video Frame	x_Man_ [dot]	y_Man_ [dot]	SD_Man_ [cm]	SD_Test_ [cm]	PSD [cm]	Passed/ Failed
1	1	S5	0:00:02.460	8.1	2.9	43.0	24.7	20.0	Passed
2	1	S6	0:00:03.125	10.6	3.0	55.1	31.6	20.0	Passed
3	1	S7	0:00:02.416	11.1	0.6	55.6	31.9	20.0	Passed
4	1	S8	0:00:01.958	7.1	1.9	36.7	21.1	20.0	Passed
5	1	S9	0:00:03.208	9.5	2.0	48.5	27.9	20.0	Passed
6	3	S10	0:00:03.750	8.5	0.0	42.5	24.4	20.0	Passed
7	3	S11	0:00:02.458	6.6	2.2	34.8	19.9	20.0	Failed
8	3	S12	0:00:03.375	7.0	0.5	35.1	20.1	20.0	Passed
9	4	S13	0:00:04.333	7.3	1.7	37.5	21.5	20.0	Passed
10	4	S14	0:00:04.291	5.7	1.8	29.9	17.2	20.0	Failed
11	4	S15	0:00:03.418	6.2	0.1	31.0	17.8	20.0	Failed
12	4	S16	0:00:04.500	6.4	3.7	37.0	21.2	20.0	Passed

**Table 3:** Comparison of the computed PSD in the proposed sokution and two reference solutions.

v_h_ [m/s]	v_r_ [m/s]	PSD [m] (Proposed)	PSD [m] (2021 [12])	PSD [m] (2012 [13])
0.25	0.0	0.428	0.330	1.256
0.25	0.5	0.756	0.578	1.508
0.25	1.0	1.084	0.827	1.813
0.25	1.5	1.412	1.075	2.168
0.25	2.0	1.740	1.324	2.573
1.60	0.0	1.659	1.680	1.806
1.60	0.5	1.987	1.928	2.196
1.60	1.0	2.315	2.177	2.636
1.60	1.5	2.643	2.425	3.126
1.60	2.0	2.971	2.677	3.666
2.50	0.0	2.480	2.580	2.175
2.50	0.5	2.808	2.828	2.655
2.50	1.0	3.136	3.077	3.185
2.50	1.5	3.464	3.325	3.765
2.50	2.0	3.792	3.577	4.395

## Data Availability

The experimental data used in this paper, including LiDAR point clouds and trajectories of the robot links, are available at https://github.com/Pecniks/lidar-robot-demo, accessed on 25 April 2023. For additional information, please contact the authors A.N., S.P. or D.P. by email.

## Supplementary Materials

The following supporting information can be downloaded at: https://www.mdpi.com/article/10.3390/s23094305/s1, Video S1: Test Scenario 1—Slow movement of the intruder towards the robot, Video S2: Test Scenario 2—Fast movement of the intruder towards the robot, Video S3: Test Scenario 3—A stationary intruder, Video S4: Test Scenario 4—An intruder approaching the robot with his arm only, Videos S5–S16: Validation tests from Table 2.

## Abbreviations

The following abbreviations are used in this manuscript:

CIP	Common industrial protocol
FK	Forward kinematics / Forward kinematic
GPS	Global positioning system
HRC	Human–robot collaboration
ICS	Intelligent control system
IMU	Inertial measurement unit
LiDAR	Light detection and ranging
PCL	Point cloud library
PSD	Protective separation distance
RGB	Red, Green, Blue
RGB-D	Red, Green, Blue – Depth
SSM	Speed and separation monitoring
TCP	Tool centre point
TIN	Triangulated irregular network
TOF	Time-of-flight
UDP	User datagram protocol
